# DESIGNING A BIOEXPERIENTIAL PROGRAM FOR SAFETY, USABILITY, AND ACCEPTABILITY WITH DYADS WITH ADVANCED DEMENTIA

**DOI:** 10.1093/geroni/igae098.2068

**Published:** 2024-12-31

**Authors:** Evan Plys, Elizabeth Rochon, Ayush Thacker, Maimouna Sy, Mirelle Phillips, Mischa Kuma, Christine Ritchie, Ana-Maria Vranceanu

**Affiliations:** Massachusetts General Hospital, Boston, Massachusetts, United States; Massachusetts General Hospital, Boston, Massachusetts, United States; Massachusetts General Hospital, Boston, Massachusetts, United States; Massachusetts General Hospital, Boston, Massachusetts, United States; Studio Elsewhere, Brooklyn, New York, United States; Studio Elsewhere, Brooklyn, New York, United States; Mass General Hospital and Harvard Medical School, Boston, Massachusetts, United States; Massachusetts General Hospital, Boston, Massachusetts, United States

## Abstract

Dyadic psychosocial interventions that engage both persons living with dementia (PLWD) and care partners (CPs) are increasingly popular. Yet, designing dyadic interventions for advanced dementia is challenging due to mismatched abilities. Here, we describe the initial development and refinement of the Toolkit for Experiential well-beiNg in Dementia (TEND) intervention with a focus on design considerations for safety, usability, and acceptability. TEND is an online bioexperiential program (e.g., storytelling, digital art, and relaxing sounds and imagery) designed to engage PLWD and CPs together, to support positive affect, relationship satisfaction, and emotional well-being. Using a multi-phasic approach consistent with user-centered design, we conducted two focus groups with clinicians (N=10), two with PLWD-CP dyads (N=7), and one with CPs only (N=4) to inform an initial prototype. Then, we conducted eight rounds of workshops with 10 PLWD-CP dyads to inform intervention refinements. Rapid data analysis was used to analyze findings and quickly inform iterative design. Focus group participants highlighted design considerations to avoid inadvertently exacerbating behavioral and psychiatric symptoms; matching the context of use; and promoting individualization, familiar stimuli, immersion, and visual clarity of the online program. After development of an initial prototype, workshop participants informed refinements to include mixed methods of engagement (i.e., voice recognition, typing, and interactive movement); instructions, tutorials, and navigation guides; directive prompts; simplified language; and pleasant sounds. Overall, the program was perceived as safe and user feedback informed refinements to promote usability and acceptability. We highlight design elements of the TEND program that were responsive to iterative user feedback.

